# Perceived stress and willingness to quit smoking among patients with depressive and anxiety disorders seeking treatment

**DOI:** 10.1002/hsr2.503

**Published:** 2022-02-24

**Authors:** Bayan Zaid Fatani, Huda Al‐Yahyawi, AbdulAziz Raggam, Mutaz Al‐Ahdal, Sukaina Alzyoud, Ahmed N. Hassan

**Affiliations:** ^1^ Department of Psychiatry King AbdulAziz Medical City Jeddah Saudi Arabia; ^2^ Department of Medicine, Psychiatry Division King AbdulAziz University Jeddah Saudi Arabia; ^3^ Faculty of Medicine University of Jeddah Jeddah Saudi Arabia; ^4^ Department of Community and Mental Health Nursing The Hashemite University Zarqa Jordan; ^5^ Campbell Family Mental Health Research Institute, Centre for Addiction and Mental Health Toronto Ontario Canada; ^6^ Department of Psychiatry University of Toronto Toronto Ontario Canada; ^7^ Departments of Pharmacology and Toxicology University of Toronto Toronto Canada; ^8^ Institute of Medical Sciences, University of Toronto Toronto Ontario Canada

**Keywords:** anxiety, depression, nicotine dependence, smoking, stress, waterpipe, willingness

## Abstract

**Rationale and objectives:**

Little are known about nicotine dependence (ND), perceived stress, and willingness to quit smoking at different treatment stages in patient with affective disorders (AD). This study aimed to evaluate the association between ND and perceived stress among patients with AD presenting with psychiatric treatment at different clinical stages (first visit or follow‐up), and in different nicotine type users (cigarette and waterpipe smokers). We also aimed to evaluate the willingness to quit smoking and its association with barriers to quitting.

**Methods:**

This cross‐sectional mixed‐method study collected quantitative and qualitative data from patients (n = 57) presenting for treatment with AD and ND at different sites in Saudi Arabia. Quantitative validated scales were used to assess the 70 of depression symptoms, anxiety symptoms, perceived stress, and ND. Qualitative questions assessed barriers to quit smoking. We used a linear regression modeling to estimate the association between ND and perceived stress as well as to estimate the association between barrier to quit and willingness to quit.

**Results:**

ND had a statistically significant association with perceived stress (odds ratio [OR]: 2.09; 95% confidence interval [CI]: 1.20‐3.63). Participants in the follow‐up group had a higher ND score than those in the first‐visit group. One of the most commonly reported barriers to quitting was using nicotine as a stress management (33.3%), which predicted positive willingness to quit (OR: 2.23; 95% CI: 1.48‐3.37; *P* < .01). Boredom was reported as a barrier in the waterpipe group more than cigarette group.

**Conclusion:**

ND has a significant association with perceived stress regardless of treatment status in patients with AD, indicating the need to evaluate smoking cessation during the early stages of treatment for patients with AD and ND. It will be critical for clinicians to offer patients with AD alternative coping mechanisms to manage stress and boredom.

## 1 INTRODUCTION

The worldwide nicotine usage is high among people with psychiatric disorders.^1^ In 2015, over 1.1 billion individuals smoked tobacco.^2^ In Saudi Arabia, the smoking prevalence in males increased from 21.4% in 2018^3^ to 23.3% in 2020.^4^ The projected prevalence of tobacco smoking in males in 2025 is 25.4%, with a difference of 2.1% from the current prevalence,^4^ reflecting one of the highest increases compared to other countries.^4^ In addition, the variety of nicotine consumption is widening, including the use of waterpipes, also called hookah or shisha, and e‐cigarettes,^5^, ^6^ among vulnerable age groups such as university students. Negative effects of nicotine, such as cancer and premature death, are well‐known.^7^ Fortunately, the negative impact of smoking can be reversed through smoking cessation.^7^

According to a previous study, one of four nicotine smokers developed affective disorders (AD).^8^ While the worldwide prevalence of depression is approximately 13%, about 17% to 49% of smokers are considered to be suffering from the same in Saudi Arabia.^9^ Similarly, anxiety disorders are common in the country.^10^ The existing literature shows a strong relationship between AD and nicotine use.^11^, ^12^ Major depression and anxiety disorders independently increase the likelihood of smoking cigarettes.^11^ Chronic nicotine use can also contribute in the development of AD.^12^ Nicotine products act as a cholinergic agonist that reduces the number of nicotine receptors,^12^ leading to low mood, while tolerance to nicotine causes withdrawal symptoms that result in dysphoria and irritability. A study conducted on animals showed that nicotine impairs fear extinction and contextual safety learning, which worsens the course of AD among the affected.^13^ This indicates the probable benefits of smoking cessation while treating AD.^14^

Willingness to quit smoking is an important step toward behavior change, being crucial for clinicians to decide on the appropriate intervention to help patients quit smoking. A study by Reddy et al showed that a direct question on the individual's thoughts about quitting smoking successfully identified their readiness to do the same.^15^ According to a study involving a multi‐country nationally representative study sample, individuals with “probable” AD and nicotine use disorder were more likely to report willingness to quit smoking than did individuals without AD^8^; however, most of those in the study sample were not clinically diagnosed and did not receive any treatment. Previous studies have reported that individuals with depression smoke in relation to their mood, and that the changing level of nicotine dependence (ND) is related to changes in their motivation to quit smoking.^16^, ^17^

Moreover, previous research has reported that perceived stress increases the odds of smoking and the severity of nicotine withdrawal.^18^, ^19^ Nicotine is a multi‐receptor substance that enhances focus, mood, and calmness within seconds of entering the alveoli in the lungs.^20^ Smoking nicotine through cigarettes or waterpipes provides a fast delivery system of the substance, which is highly addictive^21^ and whose effects last for about 10 minutes; however, these effects rapidly transform to withdrawal symptoms of irritability, feeling tense and impatient, agitation, and worsened depressive or anxiety symptoms that are only alleviated by another nicotine dose. These withdrawal symptoms are probably exaggerated by the presence of increased systemic oxidative stress.^22^ The increased systemic oxidative stress is usually caused by issues such as having highly processed food (a major public health issue in Saudi Arabia), but this stress can also lead to heightened withdrawal symptoms of nicotine.^22^ Therefore, perceived stress could correspond to the perception of discomfort in the form of nicotine withdrawal.^23^

Little is known about the association between ND and perceived stress at different levels of clinical presentation and across different nicotine delivery systems. Patients with high ND could have high perceived stress that may affect the treatment of depressive and anxiety symptoms, especially for those who have already initiated treatment. Furthermore, it is unclear whether the willingness to quit smoking and barriers to quitting differ across various clinical presentations in the treatment of AD, and whether the willingness and barriers are different in other forms of nicotine delivery such as waterpipe smoking.

This study aimed to evaluate the association between ND and perceived stress among patients with AD presenting with psychiatric treatment at different clinical stages (first visit or follow‐up), and in different nicotine type users (cigarette and waterpipe smokers). We also aimed to evaluate the willingness to quit smoking and its association with barriers to quitting. We explored intended action to quit smoking, and quitting methods preference among these patients at different clinical stages, and in different nicotine type users.

## 2 METHODS

This study utilized a mixed‐method design (convergent design) to collect both quantitative and qualitative data. This was a fixed mixed methods design as all the quantitative and qualitative methods were planned at the start of the project. We used this type of design (ie, convergent design) for expansion of the results and to extend the depth and range of inquiry to answer our research questions as recommended by other researchers.^24^ Data on barriers to quit smoking, intended action to quit and preferred methods of quitting smoking could not be captured by our quantitative questions.

The study team approached outpatients at four psychiatric treatment hospitals in Jeddah, Saudi Arabia. This was a convenient sample that was chosen using non‐probability sampling. Healthcare practitioners referred their interested patients as well. To determine the sample size, we continued asking the qualitative questions until we reached saturation (no more new information reported). We also followed Hair's recommendation for 10 participants per measurement for the quantitative data.^25^ The study was conducted between December 2019 and September 2020. The inclusion criteria were as follows: (a) must be at least 18 years of age; (b) fluent in Arabic; (c) diagnosed by a healthcare practitioner (ie, psychiatrists) as having an AD (depressive [ie, major depression disorder] or anxiety disorder [ie, generalized anxiety disorder]) according to the diagnostic and statistical manual of mental disorder (DSM 5)^26^; (d) self‐identified as a current daily smoker; and (e) either at the first visit or stable follow‐up (defined later). The exclusion criteria were as follows: (a) diagnosed by a healthcare practitioner as having schizophrenia or any schizoaffective, bipolar, or psychotic disorder; (b) currently using any smoking cessation method.

Patients who were daily smokers and identified by their clinicians as having AD, except bipolar disorder, were invited to participate in the study. This study was approved by the Research Ethics Board of the King Abdulaziz University Hospital. All participants provided informed consent for taking part in the study.

The following scales were used to assess depressive and anxiety symptoms and perceived stress (primary outcome): Patient Health Questionnaire (PHQ‐9), Hamilton Anxiety Rating Scale (HAM‐A), and Perceived Stress Scale (PSS), respectively. PHQ‐9 and HAM‐A were chosen due to their ability to measure the severity of depressive and anxiety symptoms in similar settings to our study.^27^, ^28^ PSS were chosen due to its ability to measure the individual's appraisal of their daily life as overwhelming or uncontrolled.^29^ We used the total scores of these scales to identify the severity. The PHQ‐9 is a self‐reported nine‐item Likert scale that is used to diagnose depression and its severity, and its Arabic version has been highly reliable and valid in a Saudi sample.^30^ The HAM‐A is a clinician‐rated 14‐item Likert scale that is used to detect the severity of anxiety, and its Arabic version has been validated in an Arabic sample.^31^ The PSS is a self‐reported 14‐item Likert scale that is used to detect perceived psychological stress, and its Arabic version was found to have good reliability and construct validity, and showed good psychometric properties in multiple Arabic samples.^32^, ^33^

“Smokers” included daily tobacco cigarette, waterpipe, or e‐cigarette smokers. Those who identified themselves in the demographic questionnaire as daily smokers (7 days per week) were asked to answer either the Fagerstrom test (for cigarette or e‐cigarette smokers) or the modified Waterpipe Tolerance Questionnaire‐Arabic version (WTQ‐A) (for waterpipe smokers). These scales were chosen due to their ability to measure ND severity in cigarette's smokers and waterpipe's smokers. The Fagerstrom test is a six‐item self‐reported Likert scale that is used to measure ND among cigarette smokers, and has been validated for use in an Arabic population.^34^, ^35^ WTQ‐A is a 36‐item self‐reported Likert scale that is used to measure ND among waterpipe smokers, and has been validated for use in an Arabic population^36^; the scores in this scale were divided into five quantiles to match the scoring of the Fagerstrom test.

Participants were also asked to answer the quitting subscale of Moziak's smoking questionnaire (quantitative part), which is a six‐item self‐reported scale.^37^ This scale was chosen as it has been validated for use in an Arabic population.^37^ Moreover, participants were presented with three written open‐ended questions to determine barriers to quitting as well as knowledge and preferences regarding smoking cessation methods (Table S1). Most of these questions have been adapted from other researchers.^38^ Interviews were individually based. Piloting of these questions were conducted in the presence of a research's team member to ensure acceptability. No participant withdraws their consent to participate in the study after signing the consent form.

“Willingness to quit” (secondary outcome) was defined by asking the patient to note their current thoughts about quitting smoking, which could be the willingness to quit in the next month, the next 6 months, the future, or not at all, as defined in a previous study.^15^ This question successfully identified readiness to quit, which is one of our outcome, in previous research.^15^

PHQ‐9, HAM‐A, and PSS were evaluated as continuous measures; a higher score on each scale indicated higher severity. “First‐visit patients” met the following criteria: presented for the first time before their pharmacological or psychological psychiatric treatment started, had at least mild depression scores (≥5 on PHQ‐9) or at least mild anxiety scores (≥8 on HAM‐A), and did not require acute admission by their assessed psychiatrist. “Follow‐up patients” were those who were presented to psychiatric treatment for at least 6 weeks after their initial visit, have started their psychiatric treatment, and did not change their medication type or dosage in the past month. All of the participants in the follow‐up group were on either selective serotonin reuptake inhibitors or selective norepinephrine reuptake inhibitors medications.

### 2.1 Statistical analysis

Categorical measures were summarized as percentages and counts, while continuous measures were summarized through means and SDs. We assessed the normality of our main outcome on perceived stress (PSS) using visual methods and the Shapiro‐Wilk test. The results showed bell‐shaped curve (*w* = 0.976; *P* = .312), thereby assuming normality of the data. We assessed our primary outcome using a linear regression analysis with perceived stress (severity score) set to be the dependent variable with adjustment for confounders that included for age, sex, and clinical visit status (first visit or follow‐up), depressive symptom, and anxiety symptoms. All these adjusted variables are known to affect perceived stress. The main independent variable was the severity score of ND.

We assessed the simple association between the first visit and follow‐up groups, and between cigarettes smokers and waterpipe smokers using *t*‐test and chi‐square analyses for continuous and categorical variables, respectively. Any statistical difference between these groups was further evaluated using linear regression model to present the odds ratio. For the secondary outcome, the dependent variable was willingness to quit, while the independent variables were perceived stress, different themes of reported barriers to quit smoking (ie, stress, no desire, habit, withdrawal symptoms, boredom, and peer‐pressure), and previous quitting attempt. The WTQ‐A scores were converted to its equivalent Fagerstrom scores for inclusion in the main effect of ND.

All tests were two‐sided, and *P*‐values less than .05 were considered statistically significant. All analyses were conducted using R software (version 3.3.0).

In the qualitative part, we focused on comparing the different themes across all participants' responses in the study sample. Following Braun and Clarke, who presented a guide on thematic analysis, the basic elements of interest in the qualitative questionnaire were preliminarily coded.^39^ Thematic analysis will allow for identifying a “shared meaning” in answers collected (eg, barriers to quit smoking).^39^ Using the six‐phase approach developed by Braun and Clarke, we explored the data, generated initial codes by two authors (A.H. and M.A.) independently, searched for themes in each code independently, iteratively discussed potential themes, and defined the agreed upon themes. Finally, these themes were then converted into quantitative counts and compared between the different groups using chi‐square analysis. Each theme was used to predict the willingness to quit smoking using linear regression. NVivo software was used to organize the codes and the themes were analyzed.

## 3 RESULTS

Table 1 presents the demographic and clinical data of the overall study sample. 31.6% of the sample was female. Most participants were cigarette smokers (70.2%) while 22.8% were waterpipe smokers. Most of the respondents (86.4%) were primary smokers (ie, they started smoking before the onset of their affective disorder) with an average smoking age at onset of 18.5 ± 3.6 years, while the average age at onset of AD was 25.3 ± 8.8 years.

**TABLE 1:** Demographics and clinical data of the overall sample (n = 57)

Age: mean (SD)	29.1 (8.2)
Females (%)	18 (31.6)
Marital status (%)	
Single	38 (66.7)
Married	9 (15.8)
Separated	3 (5.3)
Divorced	6 (10.5)
Widowed	1 (1.8)
Participants with children (%)	19 (33.3)
Employment (%)	45 (78.9)
Participants with psychoactive medications (%)	29 (50.9)
Nicotine smoking type (%)	
Cigarettes only	40 (70.2)
Waterpipe only	13 (22.8)
Both	4 (7.0)
Participants perceived to be able to quit (%)	34 (59.6)
Willingness to quit (%)	
Not at all	12 (21.1)
In the future	29 (50.9)
In the next 6 months	8 (14.0)
In the next month	8 (14.0)
Secondary smokers (smoking onset after mood onset) (%)	6 (13.6)
Number of smoked cigarettes: mean (SD)	18.9 (11.2)
Number of quit attempts: mean (SD)	1.3 (1.5)
Smoking age at onset: mean (SD)	18.5 (3.6)
Smoking duration: mean (SD)	11.7 (8.4)
Fagerstrom score: mean (SD)	5.7 (2.2)
Waterpipe scale score: mean (SD)	66.3 (20.8)
Mood age at onset: mean (SD)	25.3 (8.8)
PHQ score: mean (SD)	12.7 (5.7)
HAM‐A score: mean (SD)	18.0 (8.6)
PSS score: mean (SD)	21.7 (6.9)

Abbreviations: HAM‐A, Hamilton Anxiety Rating Scale; PHQ‐9, Patient Health Questionnaire; PSS, Perceived Stress Scale.

### 3.1 The association between ND and perceived stress

The average Fagerstrom test score was 5.7 ± 2.2, which is classified by the National Institute of Drug Abuse^40^ as medium to high ND. ND had a statistically significant association with perceived stress (odds ratio [OR]: 2.09; 95% confidence interval [CI]: 1.20‐3.63) after controlling for age, sex, depressive symptoms, anxiety symptoms, and clinical visit status. The higher the ND scores, the higher the perceived stress in our sample.

#### 3.1.1 First clinical visit vs follow‐up clinical visit

Table 2 demonstrates the comparison of demographic and clinical data between the first clinical visit and follow‐up. There were no differences in demographics between the two groups. As expected, the anxiety scores were lower in the follow‐up group than in the first visit group (14.5 ± 7.6 vs 20.9 ± 8.4, respectively). However, there was no statistically significant difference between the two groups in the depression and stress scores.

**TABLE 2:** Demographics and clinical data of first‐time and follow‐up (stable group) visitors for psychiatry assessment

Demographic and clinical data	Follow‐up clinical visit (n = 26)	First clinical visit (n = 31)	*P*‐value
Age: mean (SD)	31.2 (10.8)	27.4 (4.6)	.08
Females (%)	8 (69.2)	10 (32.3)	1.00
Marital status (%)			.84
Single	17 (65.4)	21 (67.7)	
Married	4 (15.4)	5 (16.1)	
Separated	1 (3.8)	2 (6.5)	
Divorced	3 (11.5)	3 (9.7)	
Widowed	1 (3.8)	0 (0.0)	
Participants with children (%)	9 (34.6)	10 (32.3)	1.00
Employment (%)	20 (76.9)	25 (80.6)	.99
Nicotine smoking type (%)			.84
Cigarettes only	19 (73.1)	21 (67.7)	
Waterpipe only	5 (19.2)	8 (25.8)	
Both	2 (7.7)	2 (6.5)	
Participants perceived to be able to quit (%)	15 (57.7)	19 (61.3)	1.00
Willingness to quit (%):			.63
Not at all	4 (15.4)	8 (25.8)	
In the future	13 (50.0)	16 (51.6)	
In the next 6 months	5 (19.2)	3 (9.7)	
In the next month	4 (15.4)	4 (12.9)	
Secondary smokers (smoking onset after mood onset) (%)	3 (16.7)	3 (11.5)	.97
Number of quit attempts: mean (SD)	1.4 (1.4)	1.3 (1.5)	.86
Smoking age at onset: mean (SD)	18.7 (3.6)	18.4 (3.7)	.80
Smoking duration: mean (SD)	14.1 (10.8)	9.7 (5.1)	.07
Fagerstrom score: mean (SD)	6.5 (2.1)	4.9 (2.1)	**.02**
Waterpipe scale score: mean (SD)	78.3 (16.2)	56.9 (19.8)	**.04**
Mood age at onset: mean (SD)	27.0 (11.8)	24.0 (5.5)	.24
PHQ score: mean (SD)	11.5 (4.5)	13.8 (6.4)	.13
HAM‐A score: mean (SD)	14.5 (7.6)	20.9 (8.4)	**.00**
PSS score: mean (SD)	20.1 (6.4)	23.0 (7.2)	.13

*Note*: Bold values denote statistically significant.

Abbreviations: HAM‐A, Hamilton Anxiety Rating Scale; PHQ‐9, Patient Health Questionnaire; PSS, Perceived Stress Scale.

**FIGURE 1:**
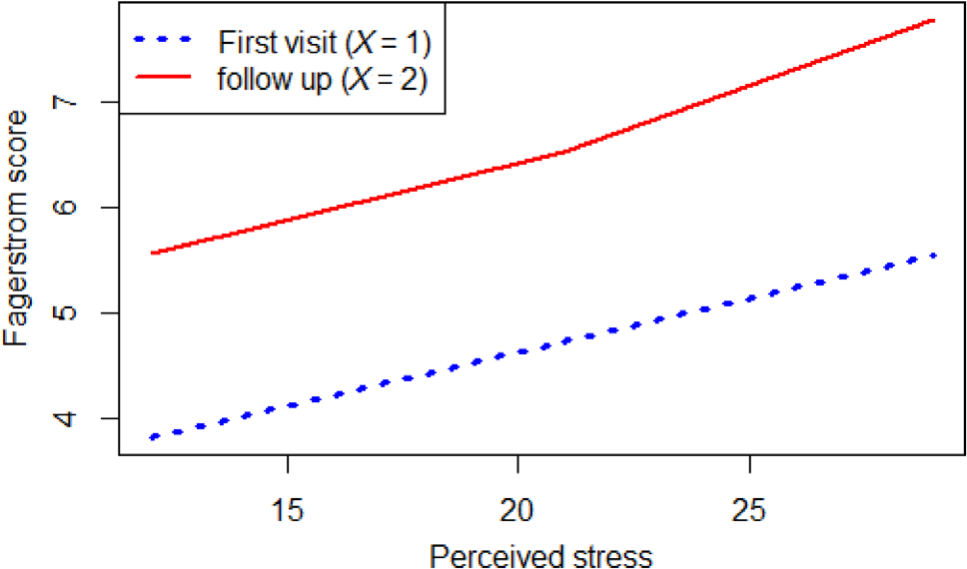
The relationship between nicotine dependence level (Fagerstrom score) and perceived stress among participants attending their first clinical visit and those in their follow‐up visit

Participants in the follow‐up group had a higher Fagerstrom average score (6.5 ± 2.1) than those in the first‐visit group (4.9 ± 2.1), which had a trend of statistically significant (*P* = .05) after controlling for age, gender, depression, anxiety, and smoking duration (OR: 6.1; 95% CI: 1.3‐27.6; *P* = .05). Figure 1 demonstrates the relationship between the Fagerstrom score and perceived stress in both groups. The waterpipe scale scores were also higher in the follow‐up (78.3 ± 16.2) than in the first‐visit group (56.89 ± 19.8; *P* = .04).

#### 3.1.2 Cigarette smoking vs waterpipe smoking

Table 3 shows a comparison of the demographic and clinical data between the two groups. Seven percent of our sample were cigarettes and e‐cigarette smokers. Employment was higher among cigarette smokers (85.4%) than among waterpipe smokers (53.8%; *P* = .04). The average ND score of the waterpipe group was 67.8 ± 22.8, which indicates medium ND. The average score of perceived stress in waterpipe smoker was 22.9 ± 4.7 while the average score of perceived stress in cigarette smokers was 21.4 ± 7.5 that was not statistically different (*P* = .51).

**TABLE 3:** Demographics and clinical data of cigarettes smokers and waterpipe (shisha) smokers

Demographic and clinical data	Cigarettes smokers (n = 44)	Waterpipe smokers (n = 13)	*P*‐value
Age: mean (SD)	28.1 (7.5)	32.6 (9.9)	.08
Females (%)	12 (27.3)	6 (46.2)	.34
Marital status (%)			.87
Single	30 (68.2)	8 (61.5)	
Married	6 (13.6)	3 (23.1)	
Separated	2 (4.5)	1 (7.7)	
Divorced	5 (11.4)	1 (7.7)	
Widowed	1 (2.3)	0 (0.0)	
Participants with children (%)	13 (29.5)	6 (46.2)	.44
Employment (%)	38 (86.4)	7 (53.8)	**.03**
First clinical visit (%)	23 (52.3)	8 (61.5)	1.00
Follow‐up duration in months mean (SD)	17.6 (40.4)	8.6 (15.3)	.22
Currently on psychoactive medications	23 (52.3)	6 (46.2)	.94
Participants perceived to be able to quit (%)	25 (56.8)	9 (96.2)	.63
Willingness to quit (%)			.42
Not at all	9 (20.5)	3 (23.1)	
In the future	21 (47.7)	8 (61.5)	
In the next 6 months	6 (13.6)	2 (15.4)	
In the next month	8 (18.2)	0 (0.0)	
Secondary smokers (smoking onset after mood onset) (%)	5 (15.2)	1 (9.0)	1.00
Number of quit attempts: mean (SD)	1.4 (1.5)	0.9 (1.3)	.27
Smoking age at onset: mean (SD)	18.4 (3.8)	19.0 (3.3)	.61
Smoking duration: mean (SD)	10.8 (7.5)	14.3 (10.7)	.21
Mood age at onset: mean (SD)	24.0 (8.6)	29.3 (8.7)	.07
PHQ score: mean (SD)	13.3 (5.5)	10.9 (6.1)	.19
HAM‐A score: mean (SD)	17.5 (8.0)	19.6 (10.4)	.44
PSS score: mean (SD)	21.4 (7.5)	22.9 (4.7)	.51

*Note*: Bold values denote statistically significant.

Abbreviations: HAM‐A, Hamilton Anxiety Rating Scale; PHQ‐9, Patient Health Questionnaire; PSS, Perceived Stress Scale.

### 3.2 Willingness and barriers to quit smoking

Over half of the sample perceived that they would be able to quit if they wanted to, while only 28% reported wanting to quit in the next 6 months. ND and perceived stress scores were not associated with willingness to quit (OR: 1.1; 95% CI: 0.95‐1.21; *P* = .36 and OR: 1.0; 95% CI: 0.97‐1.1; *P* = .67, respectively) possibly because 72% of our sample had little interest in quitting.

**FIGURE 2:**
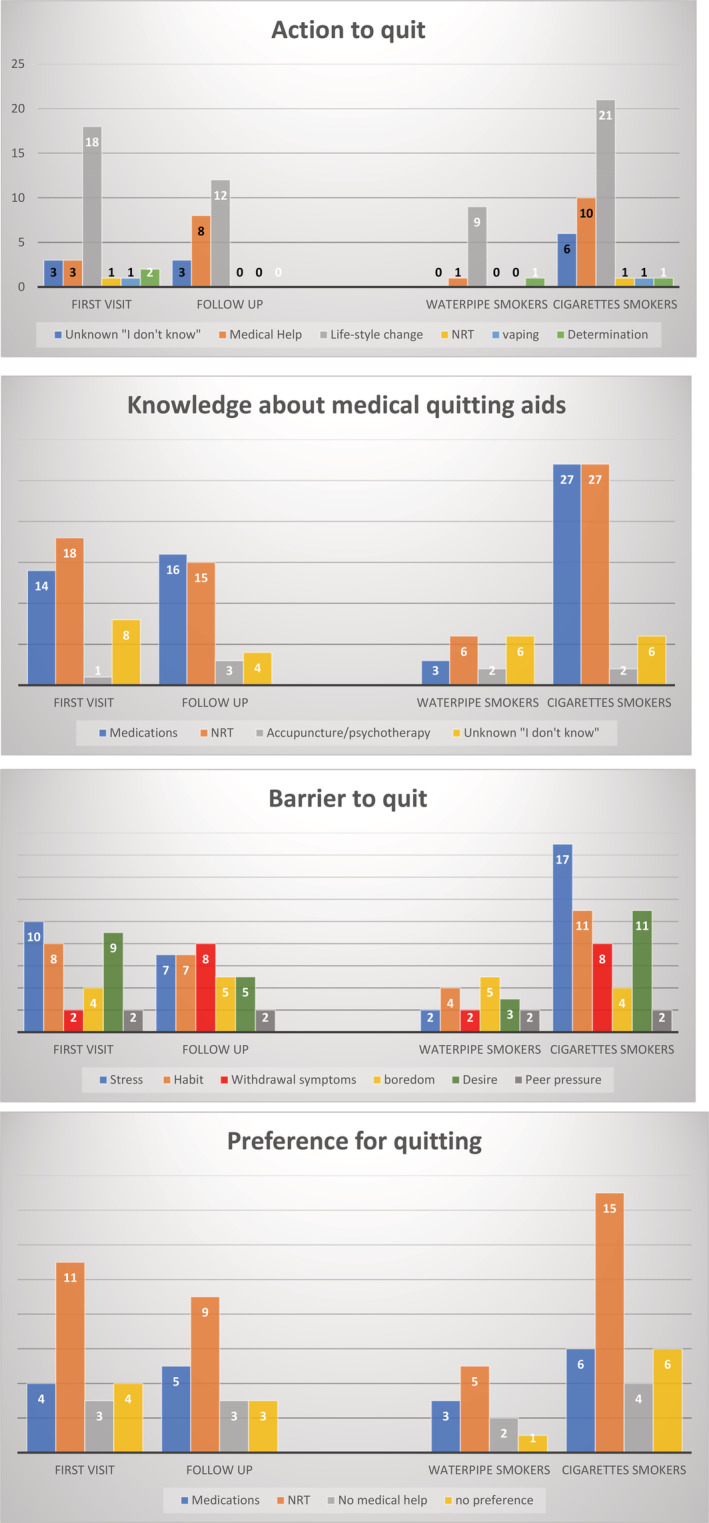
Summary and frequency of themes that have been reported to describe the barriers to quit smoking, knowledge of smoking cessation aids, intended action to quit smoking, and preference. NRT, nicotine replacement treatment

Figure 2 describes the themes of barriers to quitting, intended action to quit, knowledge of the cessation of medical aid, and preference in the method of quitting. The most reported barriers to quitting were stress management, difficulty in breaking a habit, and no desire to quit (33.3%, 25.9%, and 25.9%, respectively). Among all the reported themes of barriers to quitting, reporting stress management as a barrier to quit predicted positive willingness to quit (OR: 2.23; 95% CI: 1.48‐3.37; *P* < .01) after controlling for age, gender, depressive symptoms, anxiety symptoms, and clinical visit status; while reported no desire for quitting predicted negative willingness to quit (OR: 0.55; 95% CI: 0.34‐0.88; *P* = .04). However, other reported barriers to quit smoking were not associated with the willingness to quit, including reported habit of smoking (OR: 0.98; 95% CI: 0.61‐1.59; *P* = .95), withdrawal symptoms (1.32; 95% CI: 0.74‐2.36; *P* = .42), boredom (OR: 0.65; 95% CI: 0.37‐1.16; *P* = .22), and peer pressure (OR: 0.94; 95% CI: 0.41‐2.15; *P* = .90).

Moreover, 52.6% reported that they would change their lifestyle if they wanted to quit, and only 19.3% stated that they would seek medical help. The number of previous quitting attempts predicted willingness to quit after controlling for age, gender, depressive symptoms, anxiety symptoms, and clinical visit status (OR: 1.3; 95% CI: 1.1‐1.4; *P* < .01).

#### 3.2.1 First clinical visit vs follow‐up clinical visit

There was no difference in the willingness to quit smoking between the first visit and follow‐up groups. Only eight participants from the follow‐up group (30.7%) were offered help to quit smoking by their physicians. Moreover, 34.6% and 22.6% of the follow‐up and first‐visit groups, respectively, expressed their intention to quit smoking in the next 6 months. More individuals in the follow‐up group (30.8%) expressed that smoking withdrawal symptoms were the main barriers to quitting than did individuals in the first‐visit group (6.5%), which was statistically significant (*P* = .04). Figure 2 summarizes other themes.

#### 3.2.2 Cigarette smoking vs waterpipe smoking

Among the waterpipe and cigarette smoker groups (including e‐cigarette smokers), 15% and 31.8%, respectively, stated that they intend to quit in the next 6 months. Boredom was a significant barrier to quitting smoking among waterpipe users. Boredom was reported a barrier to quitting for 38.5% and 9.1% of waterpipe and cigarette smokers, respectively, which was statistically significant (*P* = .03). Among waterpipe and cigarette smokers, 46.2% and 13.6%, respectively, were unaware of the available smoking cessation medical aid (*P* = .03).

## 4 DISCUSSION

To the best of our knowledge, this is the first study to examine the association between ND and perceived stress in patients with AD at different clinical visit statuses and different smoking types. Furthermore, this is also the first study to report willingness to quit smoking and barriers to quitting among patients with AD in Saudi Arabia, and in different groups classified by clinical visit status and smoking type.

Our results showed that individuals with high ND perceive high stress regardless of their treatment status and tobacco smoking types. This finding is consistent with a previous study that showed higher perceived stress among heavy smokers than among light smokers.^41^ Our study adds that even patients treated for their AD had high perceived stress. In fact, patients receiving treatment for their depressive and anxiety disorders had a higher severity of ND than patients who had not yet received any treatment (first visit). This is consistent with a previous report showing that individuals with AD smoke in response to improvement or worsening of their mood.^42^ This is concerning because it could mean that tobacco smoking is obstructing the full remission of depressive and anxiety symptoms by adding more systemic oxidative stress. This challenges the idea that smoking cessation would worsen depressive and anxiety symptoms. Our results support other studies indicating that smoking cessation is associated with improvements in depressive and anxiety symptoms.^43^ Our results show that smoking cessation should be implemented as soon as possible to prevent further ND. This needs to be confirmed in longitudinal trials.

The association between ND and perceived stress could be explained by the biological effect of nicotine. High ND can cause withdrawal symptoms, which include craving and negative affect that cause stress. Smoking and stress affect each other in a closed loop, and it has been shown that perceived stress is a predictor of delay‐discounting, leading individuals to act impulsively and smoke more cigarettes.^41^ This perceived stress may be enhanced due to the increased systemic oxidative stress caused by highly processed foods commonly available in Saudi Arabia. Psychosocial and environmental stressors can also contribute to heightened systemic oxidative stress perceived as stress This hypothesis of the effect of withdrawal symptoms on stress is also supported in our data as more participants in the follow‐up group considered withdrawal symptoms as the main barrier to quitting smoking than did the first visit group, which probably reflects higher ND. This is also applicable to waterpipe smokers who showed higher ND during follow‐up compared to their first visit. It may be of benefit for clinicians to educate their patients on the relationship between high ND, withdrawal, and the effect on stress.

Less than one‐third of the sample was willing to quit within 6 months, which indicates an overall low desire. This could explain why the reported willingness to quit was not predicted by ND or perceived stress, and did not differ according to clinical status or smoking type, suggesting that smokers might not be ready to quit even if their mood symptoms are under control.^44^ However, acknowledging stress management as a barrier positively predicted willingness to quit. This is consistent with the guideline recommendations stating that to facilitate quitting, clinicians should direct the attention of smokers to individual factors such as stress.^45^ It is possible that when patients are aware of using smoking as a coping mechanism to manage stress, they become motivated to quit smoking to find an alternative coping strategy. The lack of desire to quit smoking in some of our sample, which predicted low willingness to quit, is in agreement with the results of a systematic review on barriers to quitting among vulnerable groups.^46^ The authors of that review found that only individuals with mental disorders expressed a lack of desire to quit as a barrier.^46^ The authors stated that a possible reason is poor alternative coping mechanisms to deal with stress.^46^ All our reported barriers (ie, stress management, boredom, peer‐pressure, withdrawal symptoms, and habit) match the barriers listed in a systematic review of 65 quantitative and qualitative studies.^46^ Our study adds that in those with no desire to quit, a possible helpful step is to highlight the relationship between stress management and the use of nicotine as their existing coping mechanism which might enhance their motivation to quit, as indicated in our results, and then to explore other possible alternative mechanisms to cope with stress. Intuitively, more attempts at quitting led to a greater willingness to quit smoking. However, less than one‐third of the follow‐up group was offered help in quitting smoking by their clinicians. Clinicians can reflect on the benefits of quitting attempts even if they cause relapse. Alternatively, clinicians can offer a goal of reducing rather than quitting as suggested by other researchers.^47^ A limited number of individuals at the first visit reported withdrawal symptoms as a barrier to quitting but this required the individual to be able to differentiate between nicotine withdrawal stress and other roots of stress. Individuals in the follow‐up group might have been more experienced in differentiating between withdrawal stress and other roots of stress. Clinicians can educate their patients on the symptoms of nicotine withdrawal and consider nicotine replacement therapies (eg, nicotine gum) to ameliorate these withdrawal symptoms. The type of smoking intervention can be chosen according to the patient's stage of treatment (eg, preparation vs cessation) which is outlined by Baker et al.^47^ Previous research showed that extended smoking intervention (ie, nicotine replacement therapies for 26 weeks) would provide extended abstinence outcomes if combined with maintenance counseling.^48^ This approach might be particularly helpful for patients with AD if counseling includes reflection on the existing stress management skills and ongoing discussion of alternative coping mechanisms.

Boredom was a common barrier to quitting for waterpipe smokers, which might be due to the higher unemployment rate among waterpipe smokers than among cigarette smokers. This is consistent with a previous study reporting that exclusive cigarette and waterpipe users have different reasons to quit because their reasons to smoke differ.^49^ It might be useful for future longitudinal studies to consider evaluating the effect of a chronic feeling of boredom on risky substance use and diet. These studies should indicate the age at onset of this risky use for preventive measures. It is also important to note that many waterpipe smokers were unaware of the available medical cessation aids. Therefore, clinicians should provide psychoeducation to waterpipe‐smoking patients with AD about the relationship between smoking, stress, and mood with orientation to the available nicotine replacement therapies. A previous study showed that incorporating positive coping mechanisms that address boredom, such as seeking social support or cognitive planning, alleviates perceived stress and mental distress.^50^ Unfortunately, we did not have information on the age at onset of e‐cigarette smoking in our sample or whether it predated or followed conventional cigarette smoking. Those individuals might have switched to e‐cigarettes to try to quit, switched to “safter” alternatives, or started with e‐cigarettes followed by both (dual use). Research has shown that e‐cigarette smokers will continue to smoke conventional cigarettes and that starting with e‐cigarettes might act as a “gateway” to conventional cigarettes.^51^ It is essential to discourage using e‐cigarettes as a method of smoking cessation until further evidence exists, and clinicians should offer the same smoking interventions to e‐cigarette smokers as they would for conventional smokers.

Our study had several limitations. First, we used self‐reported data that depended on participants' memory and recall abilities. Second, the sample size was limited due to the current pandemic of coronavirus disease (COVID‐19). We encourage future studies to investigate the effect of ND on mood using longitudinal study designs. The sampling was not chosen using random probability sampling which limits the ability of the findings to reflect the prevalence of perceived stress or willingness to quit among patients with AD. All the patients in our study were living in Saudi Arabia which could limit the generalization of the results. As mentioned previously, the wide availability of processed food could have led to increased systemic oxidative stress, which can exacerbate the withdrawal symptoms associated with nicotine use. Nevertheless, the results showed a significant association between ND and perceived stress as well as a significant prediction of multiple barriers with willingness to quit smoking, which warrants further investigation.

## 5 CONCLUSION

Our study demonstrated that ND has a significant association with perceived stress regardless of treatment status in patients with AD. Moreover, using nicotine as stress management, difficulty parting with the habit of smoking, and having no desire to quit were the main barriers to quitting smoking. Furthermore, our results indicated that patients with longer treatment periods may have higher levels of ND. Our findings call for the need to evaluate smoking cessation during the early stages of treatment for patients with ND, who have AD. Clinicians must assess ND during the first stage of treatment for depressive and anxiety disorders. Clinicians must educate their patients on the effect of high ND, and the effect of withdrawal symptoms on stress. It will also be critical to offer patients with AD alternative coping mechanisms to manage stress. Last, awareness of available medical options for quitting smoking should be emphasized among waterpipe smokers.

## FUNDING

This research did not receive any specific grant from funding agencies in the public, commercial, or not‐for‐profit sectors.

## CONFLICT OF INTEREST

None.

## AUTHOR CONTRIBUTIONS

Conceptualization: Bayan Fatani, AbdulAziz Raggam, Mutaz Al‐Ahdal, Ahmed N. Hassan

Formal Analysis: Mutaz Al‐Ahdal, Ahmed N. Hassan

Writing – review and editing: Bayan Fatani, AbdulAziz Raggam, Mutaz Al‐Ahdal, Ahmed N. Hassan

Writing –original draft: Bayan Fatani, AbdulAziz Raggam, Mutaz Al‐Ahdal, Ahmed N. Hassan, Sukaina Alzyoud

All Authors have read and approved the final version of the manuscript. Any additional data can be requested from the corresponding author. Ahmed Hassan accepts full responsibility for the accuracy and integrity of the data provided.

## TRANSPARENCY STATEMENT

The authors affirms that this manuscript is an honest, accurate, and transparent account of the study being reported and no important aspects of the study have been omitted.

## Supporting information


**Table S1** Qualitative questions.
