# Supplementary material for: Perceived stress and willingness to quit smoking among patients with depressive and anxiety disorders seeking treatment
Source: Health Sci Rep. 2022 Feb 24;5(2):e503. doi: 10.1002/hsr2.503 (PMC8867423; doi:10.1002/hsr2.503)
Supplement: Supplementary file 1 — Table S1 Qualitative questions. [file HSR2-5-e503-s001.docx]

**Supplementary Table 1.** Qualitative questions.

| Qualitative questions for participants |
| --- |
| 1. What is stopping you from quitting smoking? |
| 1. If you decided to quit, what would you do? |
| 1. What are the smoking cessation aids that you are familiar with? Which of them do you prefer? |
